# Longitudinal assessment of psychological distress and its determinants in a sample of firefighters based in Montreal, Canada

**DOI:** 10.3389/fpsyg.2024.1303063

**Published:** 2024-02-15

**Authors:** Filippo Rapisarda, Stéphane Guay, Isabelle Ouellet-Morin, Suzie Bond, Steve Geoffrion

**Affiliations:** ^1^Research Center, Montreal University Institute of Mental Health, Montreal, QC, Canada; ^2^Département de Psychiatrie et d’Addictologie, Faculté de Médecine, Université de Montréal, Montreal, QC, Canada; ^3^École de Criminologie, Faculté des Arts et des Sciences, Université de Montréal, Montreal, ON, Canada; ^4^Département de Sciences Humaines, Lettres et Communications, Université TÉLUQ, Quebec City, QC, Canada; ^5^École de Psychoéducation, Faculté des Arts et des Sciences, Université de Montréal, Montreal, QC, Canada

**Keywords:** firefighters, longitudinal design, mental health, depressive symptoms, post traumatic symptoms, anxiety symptoms, smartphone app, determinants

## Abstract

**Introduction:**

Firefighters face elevated risks of common mental health issues, with distress rates estimated at around 30%, surpassing those of many other occupational groups. While exposure to potentially traumatic events (PTEs) is a well-recognized risk factor, existing research acknowledges the need for a broader perspective encompassing multidimensional factors within the realm of occupational stress. Furthermore, this body of evidence heavily relies on cross-sectional studies. This study adopts an intensive longitudinal approach to assess psychological distress and its determinants among firefighters.

**Methods:**

Participants were recruited from 67 fire stations in Montreal, Canada, meeting specific criteria: full-time employment, smartphone ownership, and recent exposure to at least one PTE, or first responder status. Subjects underwent a telephone interview and were directed to use an app to report depressive, post-traumatic, and generalized anxiety symptoms every 2 weeks, along with work-related stressors, social support, and coping styles. Analyses involved 274 participants, distinguishing between those exceeding clinical thresholds in at least one distress measure (the “distressed” subgroup) and those deemed “resilient.” The duration and onset of distress were computed for the distressed group, and linear mixed models were employed to evaluate determinants for each psychological distress variable.

**Results:**

Clinical psychological distress was observed in 20.7% of participants, marked by depressive, post-traumatic, and anxiety symptoms, often within the first 4-week reference period. Contextual factors (operational climate, social support, solitude) and individual factors (coping style, solitude and lifetime traumatic events in private life) exhibited more significant impacts on psychological distress than professional pressures within the firefighters’ work environment.

**Discussion:**

This study reports lower rates of psychological distress than previous research, possibly attributable to sample differences. It highlights that reported symptoms often represent a combined and transient layer of distress rather than diagnosable mental disorders. Additionally, determinants analysis underscores the importance of interpersonal relationships and coping mechanisms for mental health prevention interventions within this worker group. The findings carry implications for the development of prevention and support programs for firefighters and similar emergency workers.

## 1 Introduction

Firefighters are an occupational group at risk of common mental health problems (Wagner et al., 2021). Post traumatic stress disorder (PTSD) has been the most studied clinical condition, with estimated rates between 4 and 56% (Meyer et al., 2012; Alghamdi et al., 2016; Theleritis et al., 2020; Wagner et al., 2021), and major depression, with estimated rates between 11 and 57% (Carey et al., 2011; Alghamdi et al., 2016; Majani et al., 2022). The wide variation in prevalence estimates across cohorts and countries may probably be due to methodological differences and sociocultural differences between studied populations. Notably, the most recent survey conducted in Canada by Carleton and colleagues (Carleton et al., 2018) showed that one-third of respondents had clinical levels of post-traumatic stress disorder (13.5%), mood disorder (22.4%), anxiety disorder (19.4%), and alcohol abuse (8.0%). Moreover, 21.3% of firefighters participating in this study had at least two comorbid clinical conditions. Such a high prevalence of psychological distress thus poses a critical issue at the epidemiological level of occupational health of this group. However, these estimates are mainly based on cross-sectional studies using self-assessment screening tools, which, although useful in providing an overview of distress levels, have the limitation of providing status estimates without indicating the impact of psychological distress over time. Additionally, the single measurement of psychological distress makes it impossible to distinguish transient from lasting clinical psychological distress likely to point to clinical diagnoses and result in more potent impact on functioning. For example, Rapisarda et al. (2023) and colleagues, estimated that although 40% of healthcare workers manifested some form of psychological distress, only one-third reported distress lasting longer than 3 weeks out of an 8-week long assessment period. Another notable exception is a study conducted by Gulliver et al. (2021) and colleagues who showed that lower prevalence of common mood and anxiety disorder in cohort of new firefighters recruits over a 3 years time (PTSD 0.5–1.2%, major depressive disorder 1.2–3.4%, and generalized anxiety disorder 0.0–1.1%). Therefore, it becomes important to establish more accurate estimates of firefighters’ mental distress while increasing knowledge about the determinants of such distress and resilience.

Occupational stress may influence the severity and course of mental health problems in firefighters and has been considered a core determinant of PTSD, depression, and substance abuse (Marmar et al., 2006; Meyer et al., 2012; Edgelow and Fecica, 2023; Serrano-Ibáñez et al., 2023). Occupational stress can be conceptualized as a process resulting from the dynamic balance of factors regarding job content and context, particularly the support received within the organization (Gulliver et al., 2021). For instance, Edgelow’s Tri-Operational-Organizational-Personal Factor Model (TROOP) (Edgelow and Fecica, 2023) clusters risk factors into three categories: operational, organizational, and personal. Operational factors pertain to the nature of the job and encompass distinctive requirements and sources of pressure confronted by public safety personnel (PSP), such as managing violent situations, feeling apprehensive about potential harm that could come to themselves or their colleagues, or encountering hostile interactions with the public while on duty, workload, safety hazards, and exposure to the risk of injury or death. Organizational factors refer to aspects of the work environment that are typically managed or significantly influenced by the employer and can either exacerbate work-related stress or promote positive mental health outcomes, job contentment, and productivity: management support, workplace culture, team climate, reciprocal support between colleagues, and personal factors. Personal factors are specific to each worker individual’s characteristics or life circumstances, including family and social connections, overall health status (including mental health), and psychological factors such as personality traits and coping skills. Coping is a dynamic regulatory process relating to someone’s cognitive and behavioral efforts to manage (reduce, minimize, master, or tolerate) internal and external demands, including work-related ones, of a person-environment transaction appraised as taxing or exceeding someone’s resources (Folkman et al., 1986). The hypothesized role of coping in the context of occupation stress related to PSP has received considerable empirical support over the last decades. For example, researchers documented that risk of developing PTSD symptoms for first responders varied according to the different coping strategies they used to manage the aftershocks of the traumatic event (Marmar et al., 2006; Meyer et al., 2012). Other factors present in firefighters’ immediate environments may also be associated with an increased risk of developing PTSD, such as receiving inadequate social support from peers, co-workers, or employers compared to those who receive satisfactory support (Marmar et al., 2006; Regehr et al., 2020; Vig et al., 2020). However, despite the number of studies identifying different determinants of distress, only a few studies attempted to model their role, relative impact on distress, and longitudinal dynamics.

Research on the psychological distress of PSPs (and firefighters in particular) would, therefore, benefit from a longitudinal approach both to correctly estimate its impact over time and to clarify the role of determinants. Repeated assessment of symptoms through diaries, telephone follow-ups, or in-person visits by professionals and management supervision in the workplace has often been used to identify high-risk workers requiring additional psychological help (Ehlers et al., 2003; McFarlane et al., 2009). However, these conventional monitoring methods have limitations as they are often used by untrained individuals, tinged with stigma and discrimination in the workplace, and subject to a lack of sensitivity to adequately detect high-risk workers and insufficient resources to provide personalized monitoring (McFarlane et al., 2009; Price et al., 2016). Today, using intensive longitudinal assessment methodologies, inspired by Ecological Momentary Assessment (Stone and Shiffman, 1994; Shiffman et al., 2008) and enabled through using smartphones as a data collection tool, enables the intensive measurement of psychological distress and its determinants (Bourla et al., 2018), reducing recall bias and modeling temporal and sequential relationships between variables. Technology, including applications (or apps) for mobile devices, may offer a more viable, confidential, and effective alternative to overcome these challenges. Digital platforms represent a promising way to reach and help at-risk workers who may be more comfortable talking about sensitive or embarrassing topics online or via an electronic device (Shiffman et al., 2008).

The present study aims to explore clinical psychological distress (PTSD, anxiety, and depression) in a sample of firefighters during a 12-week monitoring period to provide insights for research and interventions. Specifically, the first objective is to describe the onset, the level and the length of the above-threshold psychological distress and the characteristics of firefighters who experience it during the monitoring time compared with those who seem to respond resiliently. The second objective is to identify and model the role of different determinants on distress and resilience: operational and contextual work-related factors, social support, and coping.

## 2 Methods

### 2.1 Design and sample

The present study adopted an intensive longitudinal assessment design to monitor psychological distress and associated factors in firefighters for 12 weeks using a mobile application. The study protocol was approved by the ethics committee of the CIUSSS de l’Est-de-l’Île-de-Montréal with the project code 2020–1898.

The study was promoted to approximatively 2.700 firefighters among 67 fire stations in Montreal (Quebec, Canada); team leaders were invited to inform their employees of the proposed project during their meetings. Inclusion criteria were as follows: working full-time, owning a smartphone, having been exposed to at least one PTE in the past few months (without specifying the length since the event occurred) or, if not, having the role of first responder, meaning that, in addition to regular fire-related duties, they may provide medical assistance in emergencies. The only exclusion criterion was being on sick leave. Financial compensation (100 CAD) was offered to each participant when all assessments were completed. Interested participants were invited to contact a study coordinator by telephone, who verified whether they met the inclusion criteria and asked for their email address to send them a copy of the consent form and a link to install the data collection application on their smartphone. Those willing to participate were asked to sign the consent form and return it to the research team by email or fax. Three hundred and three firefighters responded to the advertisement and asked to participate in the research. Of these, 10 were excluded from the screening, and another 19 dropped out of the study. Therefore, analyses were conducted on a sample of 274 participants.

### 2.2 Data collection and instruments

For participants enrolled in the study, the coordinator scheduled a telephone appointment with a trained mental health assessor at the beginning of the project to assess mental health status, including PTEs experience, using an adaptation of the MINI International Neuropsychiatric Interview 7.0.2 and to collect lifetime traumatic events through the Life Events Checklist and guide the participant through all the questionnaires via the app. Assessors were psychology or psychoeducation students trained and supervised by psychologists working at the Centre d’Étude sur le Trauma (Trauma Studies Center).

At the selected times, the app sent a notification to the participants inviting them to self-assess risk/protective factors or psychological distress on their smartphone. If the participant does not answer any of the questions, the application sends a reminder every 12 h until the questions are completed. Data collection was designed to emphasize the temporal relationship between psychological distress and its proximal determinants; thus, proximal factors (i.e., job stress, social support, and coping) were collected at weeks 1, 3, 5, 7, 9, and 11, whereas psychological distress levels were collected at weeks 0, 2, 4, 6, 8, 10, and 12. Finally, following the completion of 12 measurement intervals with the smartphone, a second telephone interview occurred (week 13). A final interview used the same questionnaires as the interview conducted at the enrollment (week 0), except for the sociodemographic data and Life Events Checklist.

All study participants completed the 13-week data collection period. Data collection occurred from September 2020 to July 2021.

#### 2.2.1 Psychological distress questionnaires

The PTSD Checklist for DSM-5 (PCL-5) (Weathers et al., 2018) is a self-report questionnaire adopted to assess the severity of post-traumatic stress disorder symptoms. For each item, the participant was asked to indicate how bothered they were by the symptom in question using a Likert scale ranging from 0 “Not at all” to 4 “Extremely.” The “global score version” of the 8-item scale was adopted (Price et al., 2014) as a brief screening tool. The 8-item scale was preferred over the 20-item scale to facilitate intensive repeated longitudinal collection (Brier and Price, 2020). A total score can be calculated by adding the scores of each item. In this study, a cutoff of 13 was adopted to establish the presence of possible clinical-level PTSD symptoms; this cutoff has been adopted in previous studies (Zuromski et al., 2019; Rapisarda et al., 2023) and, according to Price and colleagues (Price et al., 2016), would demonstrate a sensitivity of 0.95 and a specificity of 0.23. The French version was adopted in the study (Ashbaugh et al., 2016), and the Cronbach’s alpha was 0.87.

The Patient Health Questionnaire – 9-item version (PHQ-9) (Kroenke et al., 2001) was used to assess the severity of depressive symptoms. Participants stated how much they had been bothered by each symptom over the past 2 weeks on a Likert scale from 0 “never” to 3 “almost every day”; a total score of 10 or more indicated moderate or severe depressive symptoms. This questionnaire has shown very good psychometric properties in the original and French versions (Carballeira et al., 2007), and Cronbach’s alpha was 0.86 for the present study.

Generalized Anxiety Disorder 7-item (GAD-7) is a self-report questionnaire assessing the severity of anxiety symptoms. Participants responded to each question by indicating the extent to which they were bothered on a 4-point Likert scale, and a total score of 10 or more indicated moderate or severe generalized anxiety symptoms, similar to PHQ-9. Validation of the French version showed excellent internal consistency (Micoulaud-Franchi et al., 2016) which was confirmed in the present study (Cronbach’s alpha = 0.87).

#### 2.2.2 Individual characteristics at baseline

Sociodemographic and occupational data were collected during an initial telephone interview, including age, gender, marital status, years of experience as a firefighter, and ranking. Some response categories were merged to facilitate data analysis and the presentation of results. For the “relational status” variable, participants living with partners were merged with those declaring themselves engaged, whereas those living alone, widowed, separated, and divorced were merged in the same category. Moreover, the response category “captain” was merged with “chief” in the professional rank item.

Research officers also examined lifetime exposure to PTEs in participants’ personal and professional lives using the Life Events Checklist for the DSM-5 (LEC-5), a 17-item self-report questionnaire used to identify PTEs in a participant’s life (Gray et al., 2004). The LEC-5 assesses exposure to 16 events known to be potentially traumatic and includes an additional item for any other extraordinarily stressful event. Participants indicate whether they experienced the event directly or witnessed or learned about it and whether the event occurred during their work in the past few months. Minimal revisions have been made to the original version, which demonstrates strong validity and reliability (Gray et al., 2004). A French version, already adopted by Rapisarda and colleagues in previous study (Rapisarda et al., 2023), was used.

#### 2.2.3 Proximal occupational, social and individual determinants

A tailored French version of the Job Stress Survey (JSS) (Marien and Michel, 2015) was adopted to assess occupational stress in emergency workers. Each item has a 9-point Likert-type frequency and intensity scale. Item scores are grouped based on sources of stress to calculate two indices: the occupational pressure index, comprising the average of the products of the intensities and frequencies of the items concerning perceived support from colleagues, superiors, and the organization, and stress related to operational work, concerning items involving direct contact with the public and rescue/protection activities. In the present study, Cronbach’s alphas are 0.90 for occupational pressure and 0.85 for operational work.

The Oslo 3-Item Social Support Scale was adopted to assess perceived social support (Kocalevent et al., 2018). Participants answer three questions with a 5-point Likert scale. The research team adapted the scale by including co-workers and supervisors as individuals who can provide social support and translated the items in French. A total score is calculated by adding the three individual scores, ranging from 3 to 14. Cronbach’s alpha for the present study was 0.78.

The short French version of the Ways of Coping Questionnaire (WCQ-S) (Bouchard et al., 1995) was used to assess and identify the coping styles used by participants. This version comprises 21 items, each ranging from 0 (not applicable and/or not used) to 3 (often used). Three scores were obtained, representing three broad coping styles: (1) avoidance/distancing, (2) social support seeking, and (3) reappraisal/problem-solving. Participants indicate how often they use each coping style to manage problems related to upsetting events at work. Cronbach’s alphas for the present study were 0.82, 0.87, and 0.89 for avoidance/distancing, social support seeking, and reappraisal/problem-solving, respectively.

### 2.3 Data analysis

Data preparation and analysis were conducted using R. Because the missing data were very few and tended to relate to only one assessment point (less than 5% for assessment point, with random distribution), they were replaced using a sample mean score without affecting the estimates at a statistically significant level.

**Figure 1:**
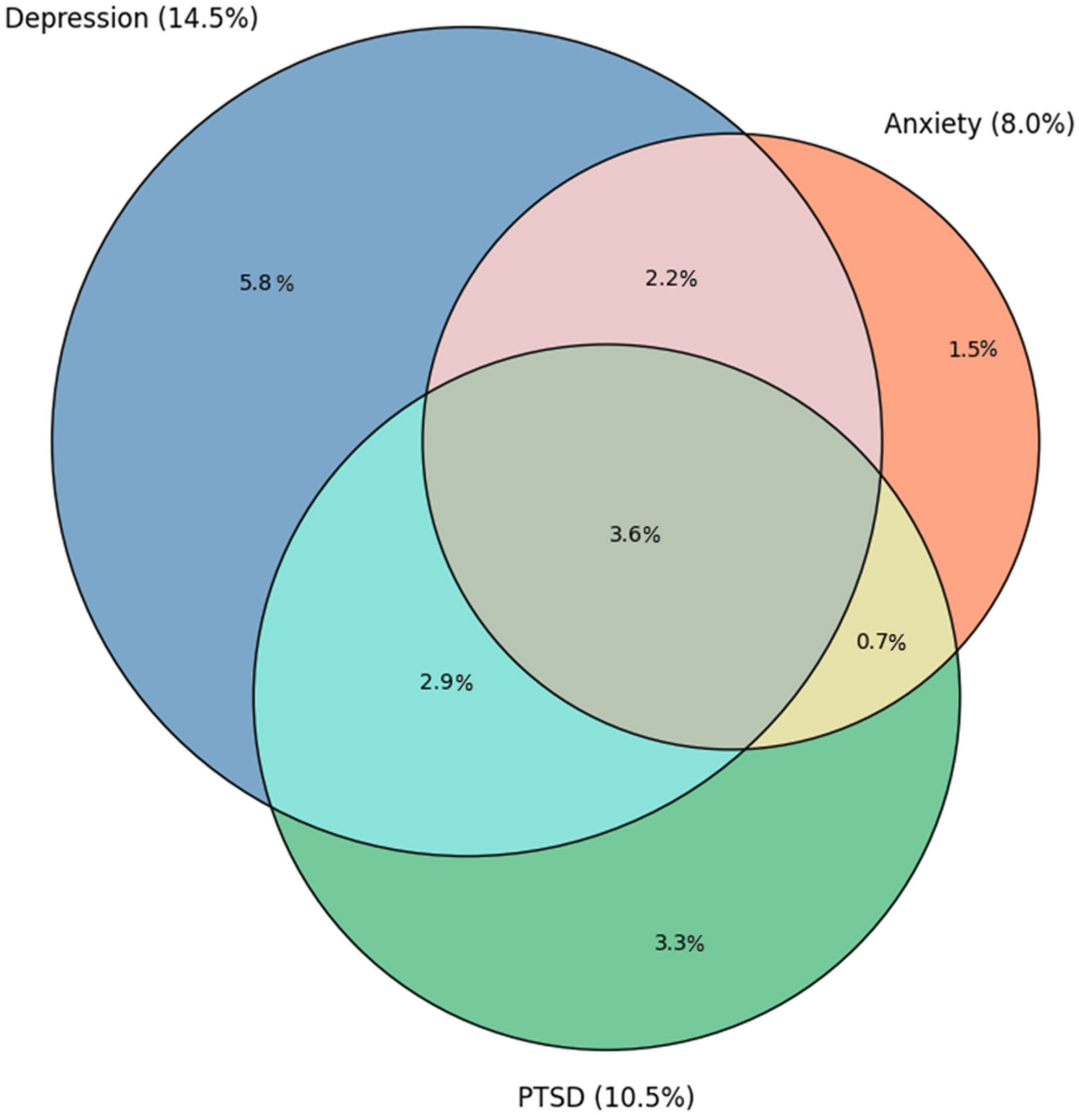
Prevalence of clinal threshold of depressive, anxiety and posttraumatic stress symptoms and their overlap. Areas in the Venn-diagram are proportionally scaled according to their estimated prevalence. Study: Longitudinal assessment of psychological distress and its determinants in a sample of firefighters in Montreal, Canada, 2020–2021.

To better describe the characteristics of firefighters who present clinical distress, participants were classified into two subgroups due to the presence of moderate and severe distress: “resilient” (RES) if distress remained below the clinical cutoffs for all three measures for the 13 weeks and “distressed” (DIS) for those scoring above the clinical cutoff in one or more measures for at least one assessment time. One of the three dimensions of distress was used as an endpoint, considering the frequent overlap that has emerged in previous studies (Hiller et al., 1989; McLaughlin et al., 2006; Gros et al., 2012; Carmassi et al., 2022). Then, descriptive statistics for all variables were computed for each subgroup (RES and DIS), and pairwise comparisons between each group for every descriptive variable were made using chi-square statistics for categorical variables and t-tests for continuous variables. For DIS subgroup participants, the number of weeks in which each exceeded the clinical cutoff (duration of distress) and the week in which this exceedance first occurred (week of onset) were calculated (Figure 1).

Linear mixed models were used to explore predictors of post-traumatic (PCL-5), anxiety (GAD-7), and depressive (PHQ-9) states at each assessment time using a repeated measures approach. Each model included baseline variables, i.e., age, relational status, rank, work experience, LEC-5 personal and work-related stressful/traumatic lifetime events and variables that were systematically collected 2 weeks before the distress questionnaires, i.e., professional pressure, and operational work from the JSS, Oslo Social Support, and WCQ, to depict temporal association between operational, contextual and individual determinants and distress. Moreover, participants’ IDs and months of data collection as random intercepts to consider the individual effects for repeated measures and the effect of the pandemic waves. The lme4 R package v.1.1–26 was used (Bates et al., 2015). Multicollinearity was assessed by calculating the variance inflation factor (VIF) for each model, but no critical VIF levels (VIF > 5) were found. In addition, a correlation matrix between predictor variables was calculated to identify possible correlations between pairs of variables. Moderate correlation among some specific predictors were retrieved, i.e.: positive correlation between age (45+) and rank (captain), positive correlation between occupational pressure and operational work and positive correlation between the three coping styles, particularly between Social Disclosure and problem solving. The matrix is reported as Supplementary material.

## 3 Results

### 3.1 Sample characteristics

Participants’ details are shown in Table 1. Almost all the participants were men (98.5%), most of whom were aged between 31 and 45 years (62.1%) and engaged in a significant relationship or married (89.3%). Approximately two out of three had the rank of firefighter (65.9%), and more than half (54.4%) had work experience between 11 and 20 years. The LEC-5 scores indicated that firefighters in our study experienced averages of 2.1 (SD = 1.6) PTEs in their private lives and 7.9 (SD = 2.1) in the work context.

**Table 1:** Comparison of clinical characteristics of resilient and distress symptoms subgroups.

Characteristic	Resilient	Moderate or severe distress	*Sig*	Overall sample
Biological sex			ns	
Woman	211 (98.1%)	46 (100.0%)		268 (98.5%)
Age			ns	
18–30	37 (17.2%)	5 (10.9%)		46 (16.1%)
31–45	127 (59.1%)	35 (76.1%)		162 (62.1%)
46 or more	51 (23.7%)	6 (13.0%)		57 (21.8%)
Relational status			*	
With partner or engaged	196 (91.2%)	37 (80.4%)		233 (89.3%)
Alone / separated / divorced	19 (8.8%)	9 (19.6%)		28 (10.7%)
Rank			ns	
Firefighter	140 (65.1%)	32 (69.6%)		172 (65.9%)
Lieutenant	38 (17.7%)	10 (21.7%)		48 (18.4%)
Captain or chief	37 (17.2%)	4 (8.7%)		41 (15.7%)
Work experience			*	
10 years or less	74 (34.4%)	11 (23.9%)		85 (32.6%)
11–20 years	88 (40.9%)	28 (60.9%)		133 (54.4%)
21 or more	53 (24.7%)	5 (17.2%)		60 (23.0%)
Life Events Checklist 5				
Personal life events, mean (SD)	2.1 (1.6)	2.5 (1.4)	ns	2.1 (1.6)
Work related events, mean (SD)	7.9 (2.2)	7.8 (1.9)	ns	7.9 (2.1)
PTE during his or her career^a^				
No specific PTE reported	128 (58.4%)	18 (32.7%)	*	146 (53.5%)
Specific PTE reported	85 (41.6%)	34 (67.3%)		119 (46.5%)
PCL-5				
Baseline assessment	2.87 (2.83)	8.3 (5.8)	***	3.5 (4.0)
Final assessment	2.06 (2.59)	10.8 (5.48)	***	2.4 (3.1)
PHQ-9				
Baseline assessment	2.70 (2.59)	11.19 (5.27)	***	3.2 (3.5)
Final assessment	2.07 (2.45)	13.00 (4.58)	***	2.8 (3.8)
GAD-7				
Baseline assessment	2.05 (2.31)	8.88 (4.7)	***	2.7 (3.1)
Final assessment	1.96 (2.22)	9.17 (4.76)	***	2.4 (3.0)
Job Stress Survey (JSS)^b^				
*Professional pressure*				
Baseline assessment	5.54 (5.69)	16.11 (11.01)	**	6.2 (6.6)
Final assessment	4.76 (6.06)	10.51 (9.47)	**	5.1 (6.5)
*Operational work*				
Baseline assessment	8.21 (5.41)	12.39 (9.35)	**	8.5 (7.0)
Final assessment	6.3 (5.96)	9.15 (7.62)	**	6.5 (7.4)
Oslo social support scale				
Baseline assessment	11.22 (2.1)	9.81 (2.93)	***	11.1 (2.2)
Final assessment	10.78 (2.39)	8.44 (2.87)	***	10.6 (2.5)
Ways of Coping Questionnaire (WCQ)^c^				
Social disclosure				
Baseline assessment	1.4 (0.5)	1.8 (0.6)	***	1.5 (0.5)
Final assessment	1.3 (0.4)	1.7 (0.6)	***	1.4 (0.5)
Reappraisal/problem solving				
Baseline assessment	1.7 (0. 6)	2.0 (0.6)	***	1.7 (0.6)
Final assessment	1.4 (0.5)	1.8 (0.6)	***	1.4 (0.5)
Avoidance / distancing				
Baseline assessment	1.3 (0.4)	2.1 (0.4)	***	1.4 (0.4)
Final assessment	1.2 (0.4)	2.0 (0.6)	***	1.3 (0.4)

Study: Longitudinal assessment of psychological distress and its determinants in a sample of firefighters based in Montreal, Canada, 2020–2021.

* = *p* < 0.05; ** = *p* < 0.01; *** = *p* < 0.001; ^a^ = information collected during the telephone interview at baseline; ^b^ = the score represents the average in the product between the intensity and frequency of stress-source elements; ^c^ = item score range from 0 (never used the coping strategy) to 3 (always used the strategy).

Two hundred and nineteen participants (79.9%) did not score above the threshold on clinical questionnaires in the 13 weeks of monitoring and were classified as resilient (RES), whereas 55 (20.7%) exceeded the clinical threshold and were classified as clinically distressed (DIS). Among the DIS participants, clinical distress was observed for an average of 6.1 weeks (SD = 4.0), and was already present at baseline in 41.8% of cases (8.7% of the total sample), decreasing nonlinearly as the distance from the baseline increased; at week 2 the onset rate was 29.1% (6.1% of the total sample) and that at week 4 was 10.9% (2.3% of the total), with a gradual decrease to 1.8% at week 13. Depressive symptoms (PHQ-9) were the most frequent above-cutoff clinical reaction (14.5%) compared with anxiety (GAD-7, 8.0%) and post-traumatic stress (PCL-5; 10.5%). Moreover, comorbidity between clinical conditions is frequent: 9.3% of participants (51.1% of the DIS subjects) scored above the cutoff in at least two conditions, and 3.6% (19.8% of the DIS subjects) showed clinically significant comorbidity between depression, anxiety, and PTSD.

Compared to the RES group, participants in the DIS group were most frequently alone, separated, or divorced (*p* < 0.05); DIS participants had a higher chance of being in the intermediate experience level. No differences between the two groups were found in lifetime traumatic experiences, assessed through the LEC-5 scores; however, participants in the DIS group reported during the baseline telephone interview higher rates of occupational PTEs compared to the RES ones. Psychological distress questionnaire and determinant scores differed significantly between the RES and DIS groups at baseline and follow-up: psychological distress (PCL-5, PHQ-9, and GAD-7), perceived stress (professional pressure and operational work), and coping styles (social disclosure, problem-solving, and avoidance/distancing) were higher in the DIS group, while social support was higher for the RES group.

### 3.2 Factors influencing firefighters’ psychological distress

Table 2 reports the results of three linear mixed models, one for each psychological distress questionnaire (PCL-5, PHQ-9, and GAD-7). Some determinants demonstrated a statistically significant influence on all three dependent variables: the association was positive for professional pressure (β_PCL-5_ = 0.12; *p* < 0.001; β_PHQ-9_ = 0.10; *p* < 0.001; β_GAD-7_ = 0.09; *p* < 0.01), positive for social support seeking (β_PCL-5_ = 0.06; *p* < 0.05; β_PHQ-9_ = 0.07; *p* < 0.01; β_GAD-7_ = 0.09; *p* < 0.001), positive for avoidance/distancing coping styles (β_PCL-5_ = 0.23; *p* < 0.001; β_PHQ-9_ = 0.19; *p* < 0.001; β_GAD-7_ = 0.23; *p* < 0.001), positive for self-reported PTEs assessed at baseline interview (β_PCL-5_ = 0.12; *p* < 0.001; β_PHQ-9_ = 0.11; *p* < 0.01; β_GAD-7_ = 0.12; *p* < 0.01), positive for personal life traumatic/stressful events assessed at baseline with LEC-5 (β_PCL-5_ = 0.11; *p* < 0.05; β_PHQ-9_ = 0.14; *p* < 0.01; β_GAD-7_ = 0.14; *p* < 0.01), and negative for perceived social support (β_PCL-5_ = −0.07; *p* < 0.01; β_PHQ-9_ = −0.06; *p* < 0.05; β_GAD-7_ = −0.06; *p* < 0.01). Additionally, some determinants showed significant association with only one distress variable: a lower experience level (0–10 years) was negatively associated with GAD-7 scores (β_GAD-7_ = −0.14; *p* < 0.01); being alone, separated, or divorced, compared to being in a relationship, was positively associated with PHQ-9 scores (β_PHQ-9_ = 0.11; *p* < 0.05); and reappraisal and a problem-solving coping style was negatively associated with PHQ-9 scores (β_PHQ-9_ = −0.07; *p* < 0.01).

**Table 2:** Linear mixed model regression predicting distress measures.

	Post-traumatic symptoms (PCL-5)				Depressive symptoms (PHQ-9)				Anxiety symptoms (GAD-7)			
	B (SE)	β	t	Sig.	B (SE)	β	t	Sig.	B (SE)	β	t	Sig.
Intercept	−0.05 (1.05)	0.00	−0.05		0.63 (1.02)	0.00	0.62		−0.21 (0.85)	0.00	−0.25	
Age (ref. (30-45))												
18–30	−0.78 (0.5)	−0.07	−1.56		−0.3 (0.50)	−0.03	−0.62		−0.17 (0.4)	−0.02	−0.43	
46 or more	−0.03 (0.61)	0.00	−0.06		−0.17 (0.60)	−0.02	−0.28		−0.29 (0.48)	−0.04	−0.59	
Work experience (ref. 10-20 years)												
0–10 years	−0.64 (0.43)	−0.08	−1.49		−0.76 (0.42)	−0.10	−1.79		−0.92 (0.34)	−0.14	−2.69	**
21 years or more	−0.25 (0.67)	−0.03	−0.37		−0.16 (0.67)	−0.02	−0.24		−0.43 (0.53)	−0.06	−0.80	
Role (ref. firefighter)												
Lieutenant	−0.12 (0.45)	−0.01	−0.26		−0.02 (0.45)	0.00	−0.05		−0.15 (0.36)	−0.02	−0.41	
Captain or chief	−0.77 (0.57)	−0.07	−1.37		−0.3 (0.57)	−0.03	−0.52		−0.21 (0.45)	−0.02	−0.46	
Relationship status (ref. engaged)												
Alone, separated or divorced	1.04 (0.51)	0.08	2.03	*	1.27 (0.51)	0.11	2.50	*	0.03 (0.41)	0.00	0.08	
Job Stress Survey												
Professional pressure	0.12 (0.07)	0.01	4.70	***	0.06 (0.01)	0.10	4.09	***	0.04 (0.01)	0.09	3.44	***
Operational work	0.02 (0.01)	0.03	1.26		0.02 (0.01)	0.03	1.6		0 (0.01)	0.00	0.12	
Oslo social support scale	−0.11 (0.04)	−0.07	−3.04	**	−0.08 (0.03)	−0.06	−2.51	*	−0.08 (0.03)	−0.06	−2.70	**
Ways of Coping Questionnaire												
Social support seeking	0.06 (0.03)	0.06	2.27	*	0.07 (0.02)	0.07	2.78	**	0.08 (0.02)	0.09	3.48	***
Reappraisal/problem solving	0 (0.02)	0.00	0.20		−0.05 (0.02)	−0.07	−2.70	**	0 (0.02)	0.01	0.26	
Avoidance / distancing	0.32 (0.03)	0.23	11.24	***	0.24 (0.03)	0.19	9.57	***	0.26 (0.02)	0.23	10.78	***
PTE reported at baseline	1.14 (0.31)	0.12	3.66	***	0.83 (0.31)	0.11	2.63	**	0.73 (0.25)	0.12	2.98	**
Life Events Checklist 5												
Personal life events	0.27 (0.12)	0.11	2.27	*	0.31 (0.12)	0.14	2.63	**	0.27 (0.09)	0.14	2.83	**
Work related events	0.06 (0.09)	0.03	0.65		0.07 (0.09)	0.04	0.84		0.05 (0.07)	0.04	0.75	

Study: Longitudinal assessment of psychological distress and its determinants in a sample of firefighters based in Montreal, Canada, 2020–2021.

* = *p* < 0.05; ** = *p* < 0.01; *** = *p* < 0.001.

## 4 Discussion

The objectives of the present study were to estimate the clinical distress levels in the population of firefighters exposed to PTEs and evaluate the influence of operational, organizational and individual determinants. An intensive longitudinal assessment design was adopted to accomplish this, requiring participants, firefighters who had experienced at least one PTE in the previous months or were first responders, to assess their emotional distress, perceived work stress, social support, and coping style every 2 weeks for a total of 13 weeks.

Regarding the first objective, 20.7% of participants experienced a clinical distress for at least 1 week, that in many cases consisted of depressive symptoms combined with anxiety and post-traumatic symptoms that frequently crossed the cutoff within the first 4 weeks of monitoring and tended to resolve by the end of the observation period. These results provide a further estimate of firefighters’ mental health problems, adding to both studies that have estimated higher rates (Carleton et al., 2018; Theleritis et al., 2020) and those that identify lower rates (Oliveira et al., 2023). some hypotheses can be developed to explain the differences with previous studies. The first concerns the composition of the sample. The present study sample comprised almost entirely men, unlike the aforementioned studies in which the sample was more balanced. Indeed, previous research showed that common mental health problems, such as PTSD and depression symptoms, are most frequently reported in women than men in the general population (Piccinelli and Wilkinson, 2000; McGinty et al., 2021) and in occupational settings (Stansfeld and Candy, 2006; Theleritis et al., 2020), reflecting differences in psychosocial risk factors, such as imbalance in power (Piccinelli and Wilkinson, 2000; McGinty et al., 2021). However, if we had a perfectly balanced sample of men and women and we adjust by applying the adjusted odds ratio found by Carleton for women vs. male firefighters (OR = 2.23), we would obtain a prevalence of overall distress of 33%, which is closer to Carleton’s estimates. Thus, since firefighting is a male-dominated profession, we believe the percentage we found is representative of this context. Another possible explanation for the difference in the estimation of psychological distress from previous studies can be attributed to the methodology adopted. Unlike the other studies mentioned, in this study the methodology involved telephone contact between participants and experimenters: we can assume that this may have deterred participation by people who, manifesting more intense psychological distress, might have preferred the more complete anonymity of an online survey. Thus, our prevalence data confirm that a significant percentage of participants suffer from a short-term combination of clinical conditions, in which depressive symptoms are observed concurrently with anxiety and post-traumatic symptoms, as found in other studies on public safety personnel (Carleton et al., 2018; Jitnarin et al., 2022) and healthcare personnel (Rapisarda et al., 2023). Therefore, our results suggest that the estimates of depressive, post-traumatic, and anxiety symptoms often reported in studies should be considered cautiously since, in most cases, these represent a combined and transient layer of distress rather than being diagnoses of true mental disorders. Additionally, these data support the partial usefulness of the hierarchical model of psychopathology (Conway et al., 2019), which groups depressive, generalized anxiety, and PTSD symptoms into the same cluster (subfactor) of “distress.” Future research should place less emphasis on diagnostic aspects and examine more closely how this transient clinical distress impacts work in terms of performance, sick leave or absence, shift and task organization, and team dynamics.

The second objective was achieved by estimating the role of different types of determinants through bivariate and multivariate analyses. The most significant finding is that organizational, social and individual factors (operational climate, social support, coping style and experience of PTEs in personal life) have a relevant impact in determining distress or resilient response in addition to operational factors (i.e., professional pressure). Moreover, our findings suggest that the relationship between occupational PTEs and distress may not be direct but mediated by other personal factor such coping style as previously found (Beaton et al., 1999; Dautovich et al., 2023), that influence the response to the single event. In our sample, each firefighter was previously exposed to an average of 7.9 work-related PTEs (assessed with the LEC-5) without differences between resilient and distressed groups, but participants in the distressed group reported a significantly higher frequency (67.3%) of PTEs during the telephone interview compared to the other group (41.6%). Thus, this suggests that clinical distress it’s not the linear outcome of a cumulative duty related PTEs, but may been conceptualize as a condition that emerge from the interplay between different risk factor and adjustment process within each event.

Thus, we found that, in our sample, the quality of the work climate and relationships among peers and with supervisors appear crucial to the mental health of firefighters, as already reported by Vig et al. (2020) and colleagues. Two theories (Stansfeld and Candy, 2006) aimed to explain the role of social support in determining mental health outcomes: the “demand–control–support theory” (Johnson and Hall, 1988; Johnson, 1989; Lee, 2019), which conceptualizes the role of social support from colleagues as a “buffer effect” able to reduce the impact of job demands and increase collective control over tasks, and the “effort-reward-imbalance” theory, (Siegrist, 1996), which states that when an imbalance exists between the effort the worker devotes to the tasks and feelings of esteem, efficacy, and integration they receive back (e.g., from colleagues or supervisors), psychological distress occurs. Notably, however, compared to others, a firefighter’s job is conducted entirely as a team, especially during more complex missions. Thus, the dimension of mutual support within the group assumes key importance regarding exposure to task-related stressors. As Aust et al. (2023) and colleagues highlight in their study on teamwork in firefighters, teamwork-related stressors (e.g., inadequate leadership) may be the psychosocial factor creating a shift in the individual appraisal of a mission that is perceived no longer as challenging but as stressful. The importance of social support for the psychological well-being of male firefighters also emerged in a study by O'Neill and Rothbard (2017) on the masculine organizational culture of firefighters, which determined that joviality and companionate love characterize the emotional culture of firefighting, a prototypically masculine one. However, in our study, social support is not limited to the work context; it also relates to the family and friendship context. Indeed, social support from families is positively associated with resilience in the general population (Ozer et al., 2003; Li et al., 2021), healthcare workers (Ortiz-Calvo et al., 2022), and emergency workers (Weiss et al., 1995; Regehr et al., 2001) after a stressful or traumatic event. Particularly, since job stress in emergency workers may spread to the family system (Regehr et al., 2005), the availability and quality of the family relationship can have a strong protective effect or, conversely, amplify distress through a maladaptive adjustment involving the whole family (Larson and Almeida, 1999; Thompson and Bolger, 1999; Regehr et al., 2005). Moreover, family problems are considered a common source of emotional stress for men (Ely and Meyerson, 2010), who may struggle with the pressure to be ideal workers as well as committed partners and parents (Humberd et al., 2014; Ladge et al., 2015; Reid, 2015).

Among individual factors affecting distress, coping, especially avoidance/distancing, is the one that is most strongly associated for all three types of symptoms (depressive, anxiety and post-traumatic); to a lesser extent, seeking social support and a lifetime history of PTEs in personal life also have a role. These findings confirm previous research on coping and psychological resources (Penley et al., 2002), including research on PTSD and firefighters (Theleritis et al., 2020; Hogg et al., 2023). In firefighters, using avoidance and distancing strategies has been associated with higher distress or PTSD (Beaton et al., 1999; Penley et al., 2002; Theleritis et al., 2020). It can be hypothesized that these two styles indicate a scarcity of specific psychological resources and a poor sense of self-efficacy and mastery regarding problem-solving and managing one’s negative mental states, so the person either turns to others or attempts to avoid the problem. The positive relationship between the coping style oriented toward seeking social support and distress that we found in our sample is less clearly explained, although it has already been observed in the literature (Penley et al., 2002). We hypothesize two possible explanations: the first is that the association is inverse; that is, people seek social support predominantly due to distress and not vice versa. The second is that seeking social support *per se* does not imply that this support is available, provided, and helpful (Penley et al., 2002). When one considers that people with greater distress in our sample complain of a worse social climate and less availability, their heavier reliance on support seeking raises questions concerning social skills and interpersonal dynamics that deserve more investigation. Moreover, the role that is attributed in the coping literature to the reappraisal/problem solving style is confirmed in the present study only as a resilience factor for depressive symptoms, but not for anxious or post-traumatic symptoms. Finally, the association between the total number of PTEs experienced lifetime and distress is well-known in the literature (Hogg et al., 2023) and can be considered a factor of increased vulnerability to stressors.

The findings of this study may have relevant implications for developing prevention and support programs for firefighters or other emergency workers. According to our results, such programs should consider three levels. The first is the work context, related to the group climate and organizational culture. At this level, intervention should be based on identifying and mediating conflicts in work teams, providing training for supervisors to improve communication with employees, and identifying toxic aspects of the culture (Wu et al., 2021). For example, Hagemann and Kluge (2013) tested a pilot team resource management intervention on firefighters that positively impacted knowledge, attitudes, and behavioral indicators. The second level is informal social support, with special reference to the family. Work-family support programs should be implemented, considering that since work-family issues are very individualized and vary between people, organizational initiatives must be sufficiently flexible and tailorable (Zhao et al., 2020) to support workers with a high level of work–family conflicts (Hammer et al., 2019). For instance, additional initiatives could be implemented, such as creating support groups among firefighter family members, especially in cases of emergency events, or proposing couples counseling. The personal/intrapsychic level is related to stress management and coping strategies. Several training interventions have been proposed to work on stress management strategies developed for healthcare staff (Alkhawaldeh et al., 2020; Buizza et al., 2020; de Wijn and van der doef, 2022). However, specific programs have been tested for police officers (Patterson et al., 2014) and critical incident management (Everly et al., 2002) that may be more suitable for firefighters. Finally, pilot studies (Coelhoso et al., 2019; Moberg et al., 2019; Stallman, 2019; Akin-Sari et al., 2022) suggest apps may be useful for not only monitoring but also preventive interventions in the work context.

### 4.1 Limitations and strengths

The study design presents several strengths that we believe will contribute to the validity of the results and the originality of the contribution. First, psychosocial determinants (sources of stress, social support, and coping) were predominantly collected (6 out of 8 measurement items) 2 weeks before the outcome questionnaires (PHQ-9, PCL-5, GAD-7). Therefore, the association found between determinants and outcomes suggests a temporal relationship: i.e. organizational and individual factors are still associated with distress 2 weeks after their assessment. Second, all instruments measuring psychosocial variables have been validated in the literature, and adopting an intensive longitudinal data collection strategy could reduce retrospective bias and increase ecological validity (Riley, 2014). Finally, the rate of missing data is extremely low, and no participants exited the study before the end of the 13 weeks. We hypothesize that this is partly due to the professional culture of firefighters, which is characterized by a high sense of duty.

At the same time, we identify some methodological limitations that need to be considered when interpreting the results and their implications. The first limitation concerns the self-reported assessment of work and private relationships and social support: in our study, social support is consistently assessed based on self-reported measurement, therefore is a “perceived support.” The real social dynamics at work and the quality of formal and informal social networks were not assessed. As already noted, low perceived support may not indicate a poor quality of available relationships but may also result from low interpersonal skills and dysfunctional personality traits. Moreover, we acknowledge that it was not possible to assess, among the effects, that of the fire station in which the participants served, as the uneven distribution of participants between sites did not allow for adequate statistical analyses. A possible avenue for future research is to use research designs that also assess context, such as multilevel designs in which to include the climate of the work unit, or to assess the views of family members and relate these data to the firefighter’s psychological distress. Another limitation relates to the assessment of PTEs. The instruments used (such as the JSS and LEC-5), although assess stressful sources (JSS) or events (LEC-5 and Mini Neuropsychiatric Interview), are not specific to assessing recent PTEs specific to the firefighting profession. However, we believe that their combination provides a fairly good proxy measure of a PTE related to the firefighting occupation. Moreover, only the JSS was suitable to repeatedly assess sources of distress during the monitoring period, but it could not be considered as a direct measure of PTEs *per se*. Another limitation already mentioned concerns the use of telephone interviews, a methodological choice that, even though it may increase the validity of some clinical measures, may have discouraged the participation of some potential participants. The anonymity provided by the online survey may facilitate self-disclosure with respect to sensitive content (Joinson, 2001) (such as that concerning mental health), and privacy issues may be particularly relevant in firefighters since their the professional culture may hinder the disclosure of psychological distress, considered as a sign of a weakness and failure (Henderson et al., 2016) Finally, it should be noted that although overall the VIF index was noncritical, the presence of some moderate correlation between some specific variables may have altered or masked the effect of some independent variables in the linear mixed models.

## 5 Conclusion

In summary, using apps to systematically monitor psychological distress and determinants is a tool that can inform research and organizations. In the present study, the app monitoring indicated that firefighters with clinical psychological distress are those who have a worse quality of work and personal relationships and try to avoid thinking about difficulties or seek social support when it is not helpful. Conversely, resilient firefighters are those who perceive relationships at work and in private life as more supportive and tend to manage problems with problem-solving strategies.

Therefore, the development of firefighter mental health prevention programs should consider different levels: those of organizational culture, informal support (especially family), and individual psychological characteristics, especially coping.

## Data availability statement

The raw data supporting the conclusions of this article will be made available by the authors, without undue reservation.

## Ethics statement

The studies involving humans were approved by CIUSSS de l’Est-de-l’Île-de-Montréal. The studies were conducted in accordance with the local legislation and institutional requirements. The participants provided their written informed consent to participate in this study.

## Author contributions

FP: Data curation, Formal analysis, Writing – original draft. SGu: Conceptualization, Funding acquisition, Writing – review & editing. IO-M: Writing – review & editing. SB: Writing – review & editing. SGe: Conceptualization, Investigation, Project administration, Supervision, Writing – original draft, Writing – review & editing.
