# Supplementary material for: Longitudinal assessment of psychological distress and its determinants in a sample of firefighters based in Montreal, Canada
Source: Front Psychol. 2024 Feb 15;15:1303063. doi: 10.3389/fpsyg.2024.1303063 (PMC10902061; doi:10.3389/fpsyg.2024.1303063)
Supplement: Supplementary file 1 [file Table_1.DOCX]

**Supplementary table.** Correlation matrix between predictor variables. Study: Longitudinal assessment of psychological distress and its determinants in a sample of firefighters based in Montreal, Canada, 2020-2021.

JSS = Job Stress survey; WCQ = Ways of Coping Questionnaire; LEC-5 = Life Events Checklist 5
